# Supplementary figures and images for: Fit-for-purpose quantitative liquid biopsy based droplet digital PCR assay development for detection of programmed cell death ligand-1 (PD-L1) RNA expression in PAXgene blood samples
Source: PLoS One. 2021 May 10;16(5):e0250849. doi: 10.1371/journal.pone.0250849 (PMC8109819; doi:10.1371/journal.pone.0250849)

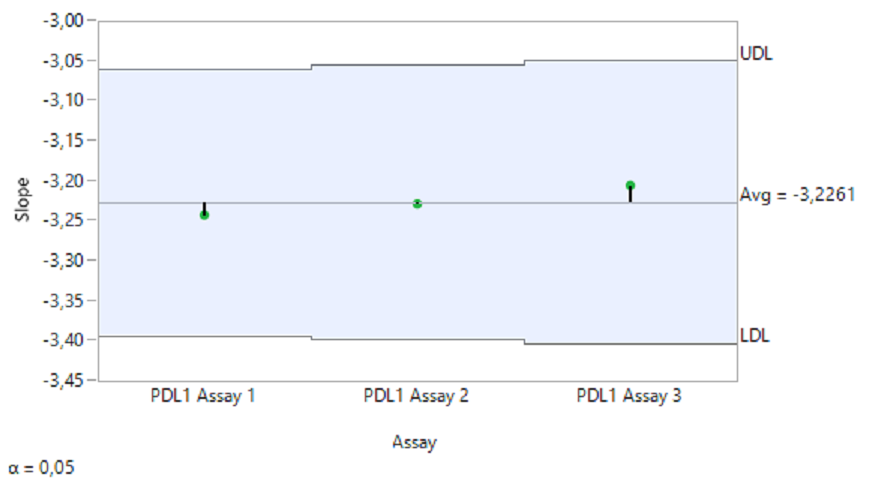

Supplement: S1 Fig — The slopes of each line of best fit from the 7-point cDNA input titration using the compare slopes function in JMP. The average slope (green dot) for each assay is shown along with the upper and lower limits. While this analysis showed no significant difference in the slopes, Assay 3 showed slightly wider upper and lower limits. (TIF) [file pone.0250849.s001.tif]
