# Supplementary material for: Fit-for-purpose quantitative liquid biopsy based droplet digital PCR assay development for detection of programmed cell death ligand-1 (PD-L1) RNA expression in PAXgene blood samples
Source: PLoS One. 2021 May 10;16(5):e0250849. doi: 10.1371/journal.pone.0250849 (PMC8109819; doi:10.1371/journal.pone.0250849)
Supplement: S1 Table — Average Cq values for four Reference genes, B2M, GUSB, TBP, and RPLP0 in A549 cells untreated and treated with IFN-γ in qPCR. (DOCX) [file pone.0250849.s002.docx]

**Supplementary Table 1:** Average Cq values for four Reference genes, B2M, GUSB, TBP, and RPLP0 in A549 cells untreated and treated with IFN-γ in qPCR.

| **Avg Cq values for Reference Genes** | | | | | | | | |
| --- | --- | --- | --- | --- | --- | --- | --- | --- |
|  | B2M Untreated | B2M + IFN-γ | GUSB Untreated | GUSB + IFN-γ | TBP Untreated | TBP + IFN-γ | RPLP0 Untreated | RPLP0 + IFN-γ |
| 100ng | 18.59705 | 14.022 | 22.7104 | 22.9625 | 23.3302 | 22.7354 | 14.9205 | 14.2712 |
| 25ng | 20.98805 | 16.766 | 24.38755 | 24.3960 | 24.9398 | 24.0703 | 17.2831 | 17.0862 |
| 6.25ng | 23.36525 | 19.051 | 26.4516 | 26.4764 | 27.2395 | 26.3763 | 19.5833 | 19.3087 |
| 1.56ng | 26.0143 | 21.48 | 28.95895 | 28.7841 | 30.107 | 29.0257 | 22.59365 | 22.1383 |
