# Supplementary material for: Fit-for-purpose quantitative liquid biopsy based droplet digital PCR assay development for detection of programmed cell death ligand-1 (PD-L1) RNA expression in PAXgene blood samples
Source: PLoS One. 2021 May 10;16(5):e0250849. doi: 10.1371/journal.pone.0250849 (PMC8109819; doi:10.1371/journal.pone.0250849)
Supplement: S2 Table — qPCR CT values for PD-L1 Taqman Assays across 7 cDNA input amounts. Cq values from qPCR assays are displayed. (DOCX) [file pone.0250849.s003.docx]

**Supplementary Table 2:** Comparison of three PD-L1 Primer/Probe Assays across 7 cDNA input amounts. Cq values from qPCR assays are displayed.

|  | **Average Cq Value** | | |
| --- | --- | --- | --- |
| **cDNA input (copies)** | **Assay 1** | **Assay 2** | **Assay 3** |
| 1000000 | 19.8364909 | 19.9722182 | 20.9263091 |
| 100000 | 22.5996091 | 23.2768545 | 24.7216727 |
| 10000 | 25.8260909 | 26.5499364 | 27.6244364 |
| 1000 | 29.2263364 | 29.6268909 | 30.7996636 |
| 100 | 32.5301818 | 32.8118727 | 34.3051636 |
| 10 | 35.4606273 | 36.30142 | 37.5461286 |
| 1 | 38.198075 | 38.094025 | 39.0532333 |
