# Supplementary material for: Fit-for-purpose quantitative liquid biopsy based droplet digital PCR assay development for detection of programmed cell death ligand-1 (PD-L1) RNA expression in PAXgene blood samples
Source: PLoS One. 2021 May 10;16(5):e0250849. doi: 10.1371/journal.pone.0250849 (PMC8109819; doi:10.1371/journal.pone.0250849)
Supplement: S3 Table — The compare slopes method resulted in no difference in the slopes of the equation of the line of best fit for our cDNA standard curve. The average, and upper and lower limits are described. (DOCX) [file pone.0250849.s004.docx]

**Supplementary Table 3:** The compare slopes method resulted in no difference in the slopes of the equation of the line of best fit for our cDNA standard curve. The average, and upper and lower limits are described.

| **Assay** | **Lower Limit** | **Group Mean** | **Upper Limit** |
| --- | --- | --- | --- |
| PDL1 Assay 1 | -3,39283 | -3,24261 | -3,05939 |
| PDL1 Assay 2 | -3,39767 | -3,22869 | -3,05455 |
| PDL1 Assay 3 | -3,40294 | -3,20557 | -3,04928 |
