# Supplementary material for: Fit-for-purpose quantitative liquid biopsy based droplet digital PCR assay development for detection of programmed cell death ligand-1 (PD-L1) RNA expression in PAXgene blood samples
Source: PLoS One. 2021 May 10;16(5):e0250849. doi: 10.1371/journal.pone.0250849 (PMC8109819; doi:10.1371/journal.pone.0250849)
Supplement: S4 Table — A. Relative quantitation with qPCR. Relative Quantitation using qPCR is measured using the 2^(-ΔΔCq) method where GUSB is used as the reference gene and A549 untreated cDNA is the reference sample. B. Relative quantitation with ddPCR. Relative Quantitation using ddPCR was calculated by first normalizing all samples to GUSB (PD-L1 copies/GUSB copies) and then normalized to A549 untreated cDNA (normalized PD-L1 copies of sample X/normalized PD-L1 copies of A549 untreated cDNA). (DOCX) [file pone.0250849.s005.docx]

**Supplementary Table 4A:** Relative Quantitation using qPCR is measured using the 2^(-ΔΔCq) method where GUSB is used as the reference gene and A549 untreated cDNA is the reference sample.

| qPCR | PD-L1 Assay 1 | PD-L1 Assay 2 | PD-L1 Assay 3 |
| --- | --- | --- | --- |
| PAXgene Blood Sample 1 | 0.63 | 0.49 | 0.55 |
| PAXgene Blood Sample 2 | 0.44 | 0.48 | 0.55 |
| PAXgene Blood Sample 3 | 0.34 | 0.30 | 0.47 |
| PAXgene Blood Sample 4 | 0.89 | 0.52 | 0.99 |
| PAXgene Blood Sample 5 | 0.52 | 0.37 | 0.43 |
| **Average PAXgene Relative Expression** | **0.56** | **0.43** | **0.60** |
| A549 untreated cDNA | 1.00 | 1.00 | 1.00 |
| A549 + IFN-γ treated cDNA | 5.22 | 7.38 | 9.37 |

**Supplementary Table 4B**: Relative Quantitation using ddPCR was calculated by first normalizing all samples to GUSB (PD-L1 copies/ GUSB copies) and then normalized to A549 untreated cDNA (normalized PD-L1 copies of sample X/ normalized PD-L1 copies of A549 untreated cDNA).

| ddPCR | PD-L1 Assay 1 | PD-L1 Assay 2 | PD-L1 Assay 3 |
| --- | --- | --- | --- |
| PAXgene Blood Sample 1 | 1.11 | 0.81 | 0.79 |
| PAXgene Blood Sample 2 | 1.89 | 1.37 | 1.26 |
| PAXgene Blood Sample 3 | 0.82 | 0.55 | 0.56 |
| PAXgene Blood Sample 4 | 1.63 | 1.32 | 1.32 |
| PAXgene Blood Sample 5 | 1.20 | 0.94 | 0.85 |
| **Average PAXgene Relative Expression** | **1.33** | **1.00** | **0.96** |
| A549 untreated cDNA | 1.00 | 1.00 | 1.00 |
| A549 + IFN-γ treated cDNA | 9.78 | 10.79 | 13.43 |
