# Supplementary material for: Fit-for-purpose quantitative liquid biopsy based droplet digital PCR assay development for detection of programmed cell death ligand-1 (PD-L1) RNA expression in PAXgene blood samples
Source: PLoS One. 2021 May 10;16(5):e0250849. doi: 10.1371/journal.pone.0250849 (PMC8109819; doi:10.1371/journal.pone.0250849)
Supplement: S5 Table — A. “Absolute” quantification for PD-L1 inn qPCR. “Absolute” quantification for PD-L1 in qPCR was determined by calculating the copy numbers based on a standard curve of the synthetic cDNA construct. This standard curve was verified in ddPCR to ensure accurate copy numbers. B. Absolute quantification for PD-L1 in ddPCR. (DOCX) [file pone.0250849.s006.docx]

**Supplementary Table 5A**: “Absolute” quantification for PD-L1 in qPCR was determined by calculating the copy numbers based on a standard curve of the synthetic cDNA construct. This standard curve was verified in ddPCR to ensure accurate copy numbers.

| qPCR | PD-L1 Assay 1 | PD-L1 Assay 2 | PD-L1 Assay 3 |
| --- | --- | --- | --- |
| PAXgene Blood Sample 1 | 1541.74 | 1656.23 | 1898.07 |
| PAXgene Blood Sample 2 | 1742.56 | 1962.57 | 2418.99 |
| PAXgene Blood Sample 3 | 1273.97 | 1308.73 | 1435.26 |
| PAXgene Blood Sample 4 | 1489.91 | 2189.44 | 1652.44 |
| PAXgene Blood Sample 5 | 1049.64 | 929.40 | 1210.52 |
| Average PAXgene Absolute Expression | 1419.56 | 1429.27 | 1723.06 |
| A549 untreated cDNA | 5187.67 | 4641.93 | 5608.56 |
| A549 + IFN-γ treated cDNA | 44606.33 | 41224.823 | 52520.37 |

**Supplementary Table 5B**: Absolute quantification for PD-L1 in ddPCR

| ddPCR | PD-L1 Assay 1 | PD-L1 Assay 2 | PD-L1 Assay 3 |
| --- | --- | --- | --- |
| PAXgene Blood Sample 1 | 3490 | 3225 | 2325 |
| PAXgene Blood Sample 2 | 4150 | 3830 | 2610 |
| PAXgene Blood Sample 3 | 3020 | 2595 | 1955 |
| PAXgene Blood Sample 4 | 2825 | 2920 | 2155 |
| PAXgene Blood Sample 5 | 1610 | 1600 | 1067 |
| Average PAXgene Absolute Expression | 3019 | 2834 | 2022 |
| A549 untreated cDNA | 4060 | 5170 | 3815 |
| A549 + IFN-γ treated cDNA | 43400 | 60950 | 56000 |
