# Supplementary material for: Fit-for-purpose quantitative liquid biopsy based droplet digital PCR assay development for detection of programmed cell death ligand-1 (PD-L1) RNA expression in PAXgene blood samples
Source: PLoS One. 2021 May 10;16(5):e0250849. doi: 10.1371/journal.pone.0250849 (PMC8109819; doi:10.1371/journal.pone.0250849)
Supplement: S6 Table — A. Inter-assay reproducibility. Inter-assay reproducibility was calculated for ddPCR (absolute quantification), and qPCR (both “absolute” quantification and relative quantification”. In each instance average values were obtained for three samples across three different runs. These values were then averaged and %CV was calculated. B. Intra-assay reproducibility. Intra-assay reproducibility was calculated based on three different runs each with five replicates. The percent CV in each run was calculated (displayed below). The average CV from each run was then averaged to get an overall intra-assay CV. (DOCX) [file pone.0250849.s007.docx]

**Supplementary Table 6A:** Inter-assay reproducibility was calculated for ddPCR (absolute quantification), and qPCR (both “absolute” quantification and relative quantification”. In each instance average values were obtained for three samples across three different runs. These values were then averaged and %CV was calculated.

| **ddPCR** | | | | | | |
| --- | --- | --- | --- | --- | --- | --- |
| **Copies/µL** |  |  |  |  |  |  |
| **PDL1 Assay 1** | Run 1 | Run 2 | Run 3 | Ave | STD DEV | CV % |
| A549 unt | 5900 | 5370 | 6885 | 6051.667 | 768.8032 | 12.70399 |
| A549 unt 1:10 | 516.5 | 545.5 | 510.75 | 524.25 | 18.62626 | 3.552934 |
| A549 + IFN- γ | 59150 | 57825 | 48600 | 55191.67 | 5746.865 | 10.41256 |
|  |  |  |  |  |  |  |
| **PDL1 Assay 2** | Run 1 | Run 2 | Run 3 | Ave | STD DEV | CV % |
| A549 unt | 6272.5 | 6020 | 7190 | 6494.167 | 615.6924 | 9.480698 |
| A549 unt 1:10 | 634.25 | 609.25 | 626.75 | 623.4167 | 12.829 | 2.057854 |
| A549 + IFN- γ | 57350 | 57925 | 52250 | 55841.67 | 3123.733 | 5.593911 |
|  |  |  |  |  |  |  |
| **PDL1 Assay 3** | Run 1 | Run 2 | Run 3 | Ave | STD DEV | CV % |
| A549 unt | 4422.5 | 4840 | 5515 | 4925.833 | 551.2845 | 11.1917 |
| A549 unt 1:10 | 480.75 | 361 | 490.25 | 444 | 72.03688 | 16.22452 |
| A549 + IFN- γ | 48825 | 46725 | 36500 | 44016.67 | 6593.763 | 14.98015 |

| **qPCR** | | | | | | |
| --- | --- | --- | --- | --- | --- | --- |
| Relative Expression |  |  |  |  |  |  |
| **PDL1 Assay 1** | Run 1 | Run 2 | Run 3 | Ave | STD DEV | CV % |
| A549 unt | 1.005868 | 1.015943 | 1.012893 | 1.011568 | 0.005166 | 0.510738 |
| A549 unt 1:10 | 1.001043 | 1.028104 | 1.260629 | 1.096592 | 0.142703 | 13.01333 |
| A549 + IFN- γ | 6.324682 | 10.08454 | 9.169922 | 8.526383 | 1.960803 | 22.99689 |
|  |  |  |  |  |  |  |
| **PDL1 Assay 2** | Run 1 | Run 2 | Run 3 | Ave | STD DEV | CV % |
| A549 unt | 1.015505 | 1.035576 | 1.014656 | 1.021912 | 0.011841 | 1.158722 |
| A549 unt 1:10 | 0.628479 | 0.836284 | 1.001685 | 0.822149 | 0.187004 | 22.74572 |
| A549 + IFN- γ | 6.47265 | 9.365367 | 9.756963 | 8.53166 | 1.793872 | 21.02606 |
|  |  |  |  |  |  |  |
| **PDL1 Assay 3** | Run 1 | Run 2 | Run 3 | Ave | STD DEV | CV % |
| A549 unt | 1.001388 | 1.02206 | 1.00325 | 1.008899 | 0.011435 | 1.133445 |
| A549 unt 1:10 | 0.828844 | 0.699612 | 0.812303 | 0.780253 | 0.070325 | 9.013126 |
| A549 + IFN- γ | 7.367454 | 8.295657 | 8.604679 | 8.089263 | 0.643917 | 7.960148 |

| **qPCR** | | | | | | |
| --- | --- | --- | --- | --- | --- | --- |
| Absolute Quantification |  |  |  |  |  |  |
| **PDL1 Assay 1** | Run 1 | Run 2 | Run 3 | Ave | STD DEV | CV % |
| A549 unt | 5598 | 3841 | 7184.78 | 5541.26 | 1672.612 | 30.18469 |
| A549 unt 1:10 | 665.2 | 525.6 | 821.25 | 670.6833 | 147.9013 | 22.05232 |
| A549 + IFN- γ | 39384.93 | 36021.9 | 58757.94 | 44721.59 | 12271.59 | 27.43996 |
|  |  |  |  |  |  |  |
| **PDL1 Assay 2** | Run 1 | Run 2 | Run 3 | Ave | STD DEV | CV % |
| A549 unt | 13073 | 7955.7 | 11041 | 10689.9 | 2576.653 | 24.10363 |
| A549 unt 1:10 | 942.9 | 886.8 | 975.2 | 934.9667 | 44.73079 | 4.784212 |
| A549 + IFN- γ | 87273 | 73953.4 | 92389.87 | 84538.76 | 9517.506 | 11.25816 |
|  |  |  |  |  |  |  |
| **PDL1 Assay 3** | Run 1 | Run 2 | Run 3 | Ave | STD DEV | CV % |
| A549 unt | 8382.3 | 6004.3 | 5477.89 | 6621.497 | 1547.449 | 23.37008 |
| A549 unt 1:10 | 765.4 | 546.52 | 486.92 | 599.6133 | 146.6354 | 24.455 |
| A549 + IFN- γ | 80214.6 | 57361.7 | 38325.47 | 58633.92 | 20973.52 | 35.77029 |

**Supplementary Table 6B:** Intra-assay reproducibility was calculated based on three different runs each with five replicates. The percent CV in each run was calculated (displayed below). The average CV from each run was then averaged to get an overall intra-assay CV.

| **ddPCR** | | | | |
| --- | --- | --- | --- | --- |
| **CV%** | **PD-L1 Assay 1 Run1** | **PD-L1 Assay 1 Run 2** | **PD-L1 Assay 1 Run 3** | **Average** |
| A549 Unt | 6.96 | 16.63 | 5.34 | 9.46 |
| A549 Unt 1:10 | 5.78 | 7.1 | 21.02 | 11.3 |
| A549 + IFN- γ | 7.59 | 4.16 | 13.68 | 8.47 |
|  |  |  |  |  |
| **CV%** | **PD-L1 Assay 2 Run 1** | **PD-L1 Assay 2 Run 2** | **PD-L1 Assay 2 Run 3** |  |
| A549 Unt | 6.44 | 8.86 | 2.2 | 5.83 |
| A549 Unt 1:10 | 6.82 | 8.42 | 10.02 | 8.42 |
| A549 + IFN- γ | 8.19 | 1.21 | 6.88 | 5.42 |
|  |  |  |  |  |
|  | **PD-L1 Assay 3 Run1** | **PD-L1 Assay 3 Run 2** | **PD-L1 Assay 3 Run 3** |  |
| A549 Unt | 6.78 | 10.99 | 9.17 | 8.98 |
| A549 Unt 1:10 | 12.27 | 8.16 | 16.94 | 12.45 |
| A549 + IFN γ | 7.41 | 6.46 | 9.84 | 7.90 |

| **qPCR Relative** | | | | |
| --- | --- | --- | --- | --- |
| **CV %** | **PD-L1 Assay 1 Run 1** | **PD-L1 Assay 1 Run 2** | **PD-L1 Assay 1 Run 3** | **Average** |
| A549 Unt | 12.18 | 20.38 | 18.67 | 17.07667 |
| A549 Unt 1:10 | 9.78 | 9.05 | 3.37 | 7.4 |
| A549 + IFN- γ | 8.62 | 12.35 | 20.71 | 13.89333 |
|  |  |  |  |  |
| **CV%** | **PD-L1 Assay 2 Run 1** | **PD-L1 Assay 2 Run 2** | **PD-L1 Assay 2 Run 3** | **Average** |
| A549 Unt | 20.88 | 30.23 | 19.63 | 23.58 |
| A549 Unt 1:10 | 9.6 | 0.86 | 7.6 | 6.02 |
| A549 + IFN- γ | 17.05 | 6.55 | 23.32 | 15.64 |
|  |  |  |  |  |
| **CV%** | **PD-L1 Assay 3 Run 1** | **PD-L1 Assay 3 Run 2** | **PD-L1 Assay 3 Run 3** | **Average** |
| A549 Unt | 6.08 | 24.11 | 9.23 | 13.14 |
| A549 Unt 1:10 | 6.71 | 21.69 | 7.86 | 12.08667 |
| A549 + IFN- γ | 13.07 | 36.39 | 11.8 | 20.42 |

| **qPCR Absolute** | | | | |
| --- | --- | --- | --- | --- |
| **CV%** | **PD-L1 Assay 1 Run1** | **PD-L1 Assay 1 Run 2** | **PD-L1 Assay 1 Run 3** | **Average** |
| A549 Unt | 11.42 | 19.34 | 17.99 | 16.25 |
| A549 Unt 1:10 | 9.16 | 8.59 | 3.25 | 7 |
| A549 + IFN- γ | 8.05 | 24.12 | 16.68 | 16.28333 |
|  |  |  |  |  |
| **CV%** | **PD-L1 Assay 1 Run1** | **PD-L1 Assay 1 Run 2** | **PD-L1 Assay 1 Run 3** | **Average** |
| A549 Unt | 19.99 | 28.58 | 19.21 | 22.59333 |
| A549 Unt 1:10 | 9.21 | 0.81 | 6.9 | 5.64 |
| A549 + IFN- γ | 18.78 | 6.8 | 21.11 | 15.56333 |
|  |  |  |  |  |
| **CV%** | **PD-L1 Assay 1 Run1** | **PD-L1 Assay 1 Run 2** | **PD-L1 Assay 1 Run 3** | **Average** |
| A549 Unt | 5.94 | 23.21 | 8.343 | 12.49767 |
| A549 Unt 1:10 | 6.55 | 20.96 | 7.13 | 11.54667 |
| A549 + IFN- γ | 17.4 | 27.33 | 10.01 | 18.24667 |
