# Supplementary material for: Fit-for-purpose quantitative liquid biopsy based droplet digital PCR assay development for detection of programmed cell death ligand-1 (PD-L1) RNA expression in PAXgene blood samples
Source: PLoS One. 2021 May 10;16(5):e0250849. doi: 10.1371/journal.pone.0250849 (PMC8109819; doi:10.1371/journal.pone.0250849)
Supplement: S7 Table — Eight samples were sent to an external service provider to confirm a successful assay transfer. ddPCR results for the service provider and internal data is listed below. (DOCX) [file pone.0250849.s008.docx]

**Supplementary Table 7:** Eight samples were sent to an external service provider to confirm a successful assay transfer. ddPCR results for the service provider and internal data is listed below.

|  | **Service Provider Run 1** | **Service Provider Run 2** | **Service Provider Average** | **EMD Serono R&D, Inc** |
| --- | --- | --- | --- | --- |
| Sample | Copies/20uL well | Copies/20uL well | Copies/20uL well | Copies/20uL well |
| A549 0.2X IFN- γ | 18460 | 20340 | 19400 | 17090 |
| A549 0.4X IFN- γ | 44880 | 46100 | 45490 | 49550 |
| A549 0.8X IFN- γ | 71800 | 76600 | 74200 | 74300 |
| A549 IFN- γ | 94200 | 94500 | 94350 | 88400 |
| PGB3 | 3080 | 2710 | 2895 | 2811 |
| PGB4 | 4400 | 4680 | 4540 | 4510 |
| A549 Unt | 8420 | 9220 | 8820 | 8980 |
| A549 0.5X Unt | 5060 | 4680 | 4870 | 3940 |
