# Supplementary material for: Fit-for-purpose quantitative liquid biopsy based droplet digital PCR assay development for detection of programmed cell death ligand-1 (PD-L1) RNA expression in PAXgene blood samples
Source: PLoS One. 2021 May 10;16(5):e0250849. doi: 10.1371/journal.pone.0250849 (PMC8109819; doi:10.1371/journal.pone.0250849)
Supplement: S8 Table — Following assay transfer to the service provider, assay validation was performed to calculate LOB, LOD, inter-assay, intra-assay, and inter-operator reproducibility. Where N/A is listed, these samples were not tested in that scenario. (DOCX) [file pone.0250849.s009.docx]

**Supplementary Table 8:** Following assay transfer to the service provider, assay validation was performed to calculate LOB, LOD, inter-assay, intra-assay, and inter-operator reproducibility. Where N/A is listed, these samples were not tested in that scenario.

|  | **Avg. PD-L1 Copies** | | **Avg. GUSB Copies** | |
| --- | --- | --- | --- | --- |
| **Sample Name** | **Scientist 1** | **Scientist 2** | **Scientist 1** | **Scientist 2** |
| NTC | 0 | 0 | 0 | 1.3 |
| NTC | 0 | 0 | 5.6 | 0 |
| NTC | 0 | 0 | 0 | 0.9 |
| Untreated A549 cell line RNA | 21580 | 22640 | 35830 | 42480 |
| Treated A549 cDNA sample 1X | 145200 | 164000 | 32740 | 39160 |
| Treated A549 cDNA sample 0.1X | 14420 | 17380 | 3250 | 3800 |
| Treated A549 cDNA sample 0.01X | 1527 | 1980 | 350 | 472 |
| Treated A549 cDNA sample 0.001X | 175 | 234 | 30 | 59 |
| Treated A549 cDNA sample 0.001X | 174 | 214 | 26 | 46 |
| Treated A549 cDNA sample 0.001X | 169 | 204 | 47 | 43 |
| Treated A549 cDNA sample 0.001X | 169 | 211 | 41 | 46 |
| Treated A549 cDNA sample 0.001X | 162 | 213 | 48 | 46 |
| Treated A549 cDNA sample 0.001X | 177 | 235 | 41 | 54 |
| Treated A549 cDNA sample 0.0001X | 19.2 | 15.4 | 2 | 8.3 |
| Treated A549 cDNA sample 0.0001X | 16.6 | 15.4 | 2.4 | 7.3 |
| Treated A549 cDNA sample 0.0001X | 19.1 | 28 | 4.7 | 8.2 |
| Treated A549 cDNA sample 0.0001X | 11.2 | 18.1 | 5.4 | 4.7 |
| Treated A549 cDNA sample 0.0001X | 18.1 | 13.6 | 3.4 | 5.4 |
| Treated A549 cDNA sample 0.0001X | 25 | 29 | 4 | 6.4 |
| USCBD_PGB_00001.3 | 1656.23 | N/A | N/A | N/A |
| USCBD_PGB_00002.3 | 1962.57 | N/A | N/A | N/A |
| USCBD_PGB_00003.3 | 1308.73 | N/A | N/A | N/A |
| USCBD_PGB_00004.3 | 1289.44 | N/A | N/A | N/A |
| USCBD_PGB_00005.3 | 929.4 | N/A | N/A | N/A |
| USCBD_PGB_00006.3 rep 1 | 841 | 1251 | 4150 | 7940 |
| USCBD_PGB_00006.3 rep 2 | 852 | N/A | 3830 | N/A |
| USCBD_PGB_00006.3 rep 3 | 768 | N/A | 3630 | N/A |
| USCBD_PGB_00007.3 rep 1 | 1028 | 1031 | 8720 | 9450 |
| USCBD_PGB_00007.3 rep 2 | 1090 | N/A | 8820 | N/A |
| USCBD_PGB_00007.3 rep 3 | 1198 | N/A | 8660 | N/A |
| USCBD_PGB_00008.3 rep 1 | 968 | 1104 | 8000 | 9400 |
| USCBD_PGB_00008.3 rep 2 | N/A | 1248 | N/A | 9120 |
| USCBD_PGB_00008.3 rep 3 | N/A | 1089 | N/A | 9890 |
| USCBD_PGB_00009.3 rep 1 | 2300 | 2230 | 11500 | 11800 |
| USCBD_PGB_00009.3 rep 2 | N/A | 1950 | N/A | 13410 |
| USCBD_PGB_00009.3 rep 3 | N/A | 2310 | N/A | 13760 |
| USCBD_PGB_00010.3 | 958 | 969 | 7110 | 7710 |
| USCBD_PGB_00011.3 | 2540 | 2580 | 8640 | 9750 |
| USCBD_PGB_00012.3 | 6250 | 5160 | 10410 | 10860 |
| USCBD_PGB_00013.3 | 26480 | 25210 | 29050 | 28970 |
| USCBD_PGB_00014.3 | 5100 | 5540 | 7980 | 8630 |
| USCBD_PGB_00015.3 | 3470 | 3320 | 10690 | 11100 |
| USCBD_PGB_00016.3 | 1661 | 1920 | 6550 | 6470 |
| USCBD_PGB_00017.3 | 988 | 1196 | 8530 | 9860 |
| USCBD_PGB_00018.3 | 1360 | 1373 | 10800 | 11610 |
| USCBD_PGB_00019.3 | 3390 | 4230 | 11010 | 15060 |
| USCBD_PGB_00020.3 | 3310 | 3770 | 10160 | 11440 |
| USCBD_PGB_00021.3 | 2760 | 3050 | 8980 | 9690 |
| USCBD_PGB_00022.3 | 1517 | 1624 | 10380 | 10860 |
| USCBD_PGB_00023.3 | 1224 | 1023 | 8190 | 8240 |
| USCBD_PGB_00024.3 | 1340 | 1471 | 8740 | 9100 |
| USCBD_PGB_00025.3 | 1722 | 1912 | 8960 | 10400 |
| USCBD_PGB_00026.3 | 1629 | 1770 | 14100 | 15750 |
| USCBD_PGB_00027.3 | 1360 | 1494 | 7460 | 8350 |
| USCBD_PGB_00028.3 | 1655 | 1750 | 12130 | 13050 |
| USCBD_PGB_00029.3 | 3360 | 3930 | 8890 | 9540 |
| USCBD_PGB_00030.3 | 2600 | 2930 | 13770 | 15590 |
| USCBD_PGB_00031.3 | 3680 | 4920 | 6660 | 8380 |
| USCBD_PGB_00032.3 | 7510 | 7420 | 9260 | 8910 |
| USCBD_PGB_00033.3 | 3260 | 3440 | 12060 | 12580 |
| USCBD_PGB_00034.3 | 2910 | 3110 | 7840 | 7970 |
| USCBD_PGB_00035.3 | 1334 | 1381 | 5970 | 5990 |
