# Supplementary material for: Fit-for-purpose quantitative liquid biopsy based droplet digital PCR assay development for detection of programmed cell death ligand-1 (PD-L1) RNA expression in PAXgene blood samples
Source: PLoS One. 2021 May 10;16(5):e0250849. doi: 10.1371/journal.pone.0250849 (PMC8109819; doi:10.1371/journal.pone.0250849)
Supplement: S1 File — (DOCX) [file pone.0250849.s010.docx]

**Supplementary Section**

Synthetic Construct Sequence:

GGCGCAACGCTGAGCAGCTGGCGCGTCCCGCGCGGCCCCAGTTCTGCGCAGCTTCCCGAGGCTCCGCACCAGCCGCGCTTCTGTCCGCCTGCAGGGCATTCCAGAAAGATGAGGATATTTGCTGTCTTTATATTCATGACCTACTGGCATTTGCTGAACGCCCCATACAACAAAATCAACCAAAGAATTTTGGTTGTGGATCCAGTCACCTCTGAACATGAACTGACATGTCAGGCTGAGGGCTACCCGACAGAGGGCCCGGCTGTTGAAGGACCAGCTCTCCCTGGGAAATGCTGCACTTCAGATCACAGATGTGAAATTGCAGGATGCAGGGGTGTACCGCTGCATGATCAGCTATGGTGGTGCCGACTACAAGCGAATTACTGTGAAAGTCAATGCCCCATACAACAAAATCAACCAAAGAATTTTGGTTGTGGATCCAGTCACCTCTGAACATGAACTGACATGTCAGGCTGAGGGCTACCCCAAGGCCGAAGTCATCTGGACAAGCAGTGACCATCAAGTCCTGAGTGGTAAGACCACCACCACATCCTCCAAATGAAAGGACTCACTTGGTAATTCTGGGAGCCATCTTATTATGCCTTGGTGTAGCACTGACATTCATCTTCCGTTTAAGAAAAGGGAGAATGATGGATGTGAAAAAATGTGGCATCCAAGATACAAACTCAAAGAAGCAAAGTGATACACATTTGGAGGAGACGTAATCCAGCATTGGAACTTCTGATCTTCAAGCAGGGATTCTCAACCTGTGGTTTAGGGGTTCATCGGGGCTGAGCGTGACAAGAGGAAGGAATGGGCCCGTGGGATGCAGGCAATGTGGGACTTAAAA
