# Supplementary material for: Fit-for-purpose quantitative liquid biopsy based droplet digital PCR assay development for detection of programmed cell death ligand-1 (PD-L1) RNA expression in PAXgene blood samples
Source: PLoS One. 2021 May 10;16(5):e0250849. doi: 10.1371/journal.pone.0250849 (PMC8109819; doi:10.1371/journal.pone.0250849)
Supplement: Attachment — Submitted filename: Response to Reviewers_02April2021_PLOSONE.docx [file pone.0250849.s011.docx]

|  | April 2, 2021 |
| --- | --- |
|  |  |

Dear Editors,

On behalf of all authors, I would like to thank you for the generous comments on our manuscript entitled “Fit-for-purpose quantitative liquid biopsy based droplet digital PCR assay development for detection of programmed cell death ligand-1 (PD-L1) RNA expression in PAXgene blood samples.” We have edited the manuscript to address these concerns.

Namely, we have corrected some formatting issues to comply with PLOS One policy, amended the funding statements and conflict of interest statements, and added or amended sections throughout the manuscript in accordance to the recommendations. Please see below for detailed responses.

First we would like to emphasize that initially we looked to develop a RT-qPCR assay, however after initial assay characterization, our RT-qPCR was not suitable for our purpose and thus we switched to a ddPCR assay. This assay, in our experiments, provided a more appropriate dynamic range and reproducibility in addition to absolute quantification.

Below please find the changes we implemented in accordance to the reviewer’s and editors suggestions:

1. Formatting was changed in several sections including increase font size of headings and sub-headings. We also added the proper affiliation to Maria Cusano which was previously missing.
2. Reference list review – We have reviewed the referenced manuscripts and did not find any which were redacted
3. Financial disclosure statement was updated to read “This study was funded by EMD Serono Inc., a part of Merck KGaA Darmstadt, Germany. EMD Serono/ Merck KGaA is the employer of all authors (with the exception of Maria Cusano, who was previously employed by EMD Serono during the time the experiments were conducted) at the time of submission. Merck KGaA/ EMD Serono is a commercial company who provided support in the form of salaries for authors DO, DW, JSG, MC, WM, TC, JS, and ZF, but did not have any additional role in the study design, data collection and analysis, decision to publish, or preparation of the manuscript. The specific roles of these authors are articulated in the ‘author contributions’ section.” and added to amended cover letter.

Likewise the competing interest statement was updated and added to amended cover letter to read “The authors declare that they have no competing interests which may influence this publication or alter our adherence to PLOS ONE policies on sharing data and materials.”

4. Author affiliations have been amended specifically, Maria Cusano affiliation was added.
5. The captions for the supporting files was added to the end of the manuscript.
6. Line 276 – Added n=35 to line 301 of revised version
7. Lines 277-281 – Removed sentences from paragraph 6.1 so the test only appears in 6.2
8. Lines 289-303 – Paragraphs 6.3,6.5 and 6.6 were combined into one paragraph as 6.4
9. Lines 347-355 – Paragraphs 7.1 and 7.2 were combined into one paragraph 7.1
10. Lines 84,124,163-165 and 356 – To describe the relative quantitation in ddPCR the method was described in new lines 387-389.
11. Line 131 – Have changed the stated value from 0.44 to 0.43 in line 129 to reflect the accurate value in Table 4A. This discrepancy was due to a rounding error.
12. Line145 – Have added data to describe the absolute expression in qPCR for the three PD-L1 assays. Edits found in lines 140-151.
13. Line 146 – We have specified in lines 301-304 that 5 samples were used for assay development while the remaining 30 were for validation.
14. Line 163-164 – We have combined the sentences to avoid repetition.
15. Table 1 and Table 2 – We have added a row at the bottom of the table to list the average values.
16. Line 177 – We added the reference for Table 2 to lines 164 and 174 in the revised version.
17. Line 232-238 – We have added lines 178-180 to help further clarify the decision to utilize Assay 2.
18. We have added references to Supporting Table 6A, 6B, 7 and 8 in the text.
19. Line 240-243 – We could not find this section reference starting with “The ddPCR offers absolute…” and therefore we have not made any edit for this comment. If necessary, please further clarify the section which should be removed, and we can happily comply.
20. Discussion was expanded to add more details and interpretation of the results.

We believe the manuscript is now suitable for publication. However, if you feel that any section or revision needs further clarification/expansion please let me know and we are happy to accommodate.

Yours,

Dennis O’Rourke

Scientist
Healthcare | Global Development | Translational Medicine | Global Clinical Biomarkers & Companion Diagnostics

**EMD Serono**

A business of Merck KGaA, Darmstadt, Germany

EMD Serono Research & Development Institute, Inc. | 45A Middlesex Turnpike | Billerica, MA 01821 | USA

E-mail: [dennis.orourke@emdserono.com](mailto:dennis.orourke@emdserono.com)

[www.emdserono.com](http://www.emdserono.com/)
